# Similar Growth Performance but Contrasting Biomass Allocation of Root-Flooded Terrestrial Plant *Alternanthera philoxeroides* (Mart.) Griseb. in Response to Nutrient Versus Dissolved Oxygen Stress

**DOI:** 10.3389/fpls.2019.00111

**Published:** 2019-02-07

**Authors:** Qiaoli Ayi, Bo Zeng, Kang Yang, Feng Lin, Xiaoping Zhang, Peter M. van Bodegom, Johannes H. C. Cornelissen

**Affiliations:** ^1^Key Laboratory of Eco-environments in Three Gorges Reservoir Region (Ministry of Education), Chongqing Key Laboratory of Plant Ecology and Resources in Three Gorges Reservoir Region, School of Life Sciences, Southwest University, Chongqing, China; ^2^Department of Systems Ecology, Faculty of Earth and Life Sciences, Institute of Ecological Science, Vrije Universiteit Amsterdam, Amsterdam, Netherlands; ^3^Institute of Environmental Sciences, Leiden University, Leiden, Netherlands

**Keywords:** nutrient versus dissolved oxygen stress, plant growth, biomass allocation, root production, root efficiency, aerenchyma channel diameter, flooding tolerance, *Alternanthera philoxeroides*

## Abstract

Terrestrial plants may experience nutrient and oxygen stress when they are submerged, and increases in flooding are anticipated with climate change. It has been well reported that plants usually shift biomass allocation and produce more roots in response to nutrient deficiency. However, it is unclear whether plants experiencing oxygen deficiency stimulate biomass allocation to roots to enhance nutrient absorption, similar to how plants experiencing nutrient deficiency behave. We investigated the responses of the terrestrial species *Alternanthera philoxeroides*, upon root flooding, to nutrient versus dissolved oxygen deficiency in terms of plant growth, biomass allocation, root production, root efficiency (plant growth sustained per unit root surface area), and root aerenchyma formation. Both nutrient and dissolved oxygen deficiency hampered the growth of root-flooded plants. As expected, plants experiencing nutrient deficiency increased biomass allocation to roots and exhibited lower root efficiency; in contrast, plants experiencing dissolved oxygen deficiency decreased biomass allocation to roots but achieved higher root efficiency. The diameter of aerenchyma channels in roots were enlarged in plants experiencing dissolved oxygen deficiency but did not change in plants experiencing nutrient deficiency. The widening of aerenchyma channels in roots could have improved the oxygen status and thereby the nutrient absorption capability of roots in low oxygen environments, which might benefit the plants to tolerate flooding.

## Introduction

Resource availability and disturbances have been regarded as the major factors driving plant functioning (Grime, 1979) and traits associated with these two factors define the range of strategies for plant growth and existence (Ackerly, 2004). The root system is of fundamental importance for a plant to deal with these two factors because it anchors the plant to its substrate, as well as takes up water and nutrients from its soil environment (Bellini et al., 2014). Root systems are known to show a high degree of plasticity in their development in response to local heterogeneity in resource availability (Ostonen et al., 2007). Under limited resource availability conditions, taking nutrient and water deficiency as examples, the response of plants is to increase the allocation to the root systems (Poorter et al., 2012). Concomitantly, roots may increase their surface area:volume ratio and root length per unit root mass (Freschet et al., 2015) to maximize the root-soil interface thereby enhancing the absorption potential (Paula and Pausas, 2011). Plants with high root surface area:volume ratio or high root length:root mass ratio usually show improved uptake for water (Eissenstat, 1991) and nutrients (Reich et al., 1998; Comas et al., 2002). Basically, when nutrients in the environment are deficient, the amount of nutrients taken up per unit root surface area of a plant decreases accordingly, and the plant growth supported per unit root surface area is reduced. If we define root efficiency as the plant mass sustained per unit root surface area, we can hypothesize that plants living in nutrient-deficient environments have lower root efficiencies than plants growing in nutrient-rich environments.

Root nutrient uptake is generally an active, energy-dependent process (Bassirirad, 2000) and regulated to some extent by ATP production (Tournaire-Roux et al., 2003). It is well known that aerobic respiration is more efficient and produces more ATP compared to anaerobic respiration (Lambers et al., 2008). Thus, energy production in plant cells largely depends on the availability of oxygen. Due to the low solubility and slow diffusion of oxygen in water (oxygen diffusion in water is 10000-fold slower than that in air) (Jackson, 1985), the oxygen content in water is low compared with that in air. Therefore, plants (especially the flooded organs of the plants) in aquatic habitats are confronted with a poor oxygen environment when compared to plants in aerial habitats. In recent decades, the occurrence of flooding events increased dramatically in the whole world. Moreover, owing to the global climate change, climate predicting models suggest the frequency and intensity of global flood risk may increase in near future (IPCC, 2012; Hirabayashi et al., 2013; Jongman et al., 2014). Due to precipitation and floods, some terrestrial plants, especially those living in flood-prone habitats, e.g., lowlands, river banks and lake shores, experience flooding conditions during parts of their life cycle. For terrestrial species, flooding causes oxygen deficiency in flooded plant parts. Oxygen deficiency is considered to greatly inhibit the uptake of nutrient and water by roots in flood-prone environments, thereby hampering the growth of flooding-sensitive species, or even inducing their death (Crawford and Braendle, 1996).

Upon flooding, some flooding-tolerant terrestrial plants tend to produce aquatic roots (Jackson and Drew, 1984), which generally have developed aerenchyma. These aquatic roots on flooded plants are capable of absorbing nutrients, as substitutes for pre-flooding produced roots which lose their function or die upon submergence (Končalová, 1990; Polthanee and Changdee, 2008; Sauter, 2013; Zhang et al., 2018). It has been clearly demonstrated that plants enhance root production to improve nutrient uptake in nutrient-deficient environments (Hutchings and John, 2004). However, it is unknown whether the oxygen availability in water affects the root production of flooded plants, and whether plants stressed by dissolved oxygen shortage would enhance root production to improve nutrient uptake, like what plants do when stressed by nutrient shortage. If the oxygen that the roots of a flooded plant need for absorbing nutrients is supplied by tissue photosynthesis or from air (especially for waterlogged or partially submerged plants having aerial organs and structures above water), it can be expected that the dissolved oxygen concentration in water will not influence the nutrient uptake of roots and consequently plant growth will not be affected. However, if the roots are able to absorb oxygen directly from the water column, we hypothesize that, with a decrease in dissolved oxygen concentration in water, flooded plants would strengthen their root production to ensure nutrient uptake. In previous research on submergence-tolerant plants, the internal oxygen status in the submerged tissues and organs of the plants was positively related to the dissolved oxygen concentration in the water column (Pedersen et al., 2006; Rich et al., 2013). Ayi et al. (2016) demonstrated recently that aquatic roots played an important role in oxygen uptake from the water column and transported the absorbed oxygen to other organs of the submerged plants.

In the real world, nutrient shortage and flooding-caused oxygen shortage are stresses often experienced by terrestrial plants. Infertile soils provide poor nutrient environment for plants, and plants growing in swamps or submerged in stagnant backwater or billabongs have to endure hypoxic conditions. Taking *Alternanthera philoxeroides* (Mart.) Griseb., a flooding-tolerant terrestrial plant (Gao et al., 2008) as a model, we studied the effects of nutrient as well as of dissolved oxygen availability on the growth, biomass allocation, and root traits of root-flooded plants. The following questions were addressed in this study: (1) Is the growth of root-flooded plants suppressed by nutrient and dissolved oxygen shortage? (2) Do root-flooded plants enhance their biomass allocation to root production similarly under nutrient and dissolved oxygen shortage? (3) Is the root efficiency of plants similarly decreased by shortage of nutrients as by dissolved oxygen? (4) Does the shortage of nutrients or of dissolved oxygen affect the formation and morphology of aerenchyma in roots similarly? The answers to these questions will expand and deepen our understanding of how plants respond to and tolerate flooding under different environmental regimes.

## Materials and Methods

### Plant Material

*Alternanthera philoxeroides* (Mart.) Griseb., a perennial terrestrial plant of the Amaranthaceae family, originates in South America, but has spread to many parts of the world and is considered an invasive species in the United States, Australia, New Zealand, Thailand, and China. This species is found to have good tolerance to submergence, upon which it usually produces a number of aquatic roots on the nodes of the submerged stem (Ayi et al., 2016). Under normal conditions, *A. philoxeroides* grows to a height of 50–120 cm, with a long, single or sparsely branched stem.

The *A. philoxeroides* plants used in this experiment were cultivated from cuttings that had been obtained from plants naturally growing on banks of Jialing River in Chongqing, SW China (29°49′N, 106°25′E). Unbranched plants with stem length of ca. 25 cm were selected and cut at the stem base. All cuttings were taken back to the laboratory immediately, healthy and vigorous cuttings were selected and used in the experiments as described below, each cutting was regarded as a plant.

### Experimental Design

Two experiments were conducted in this study to investigate the responses of root-flooded *A. philoxeroides* plants to nutrient versus dissolved oxygen shortage, one with nutrient treatments, the other with dissolved oxygen treatments. 30 plant cuttings were used in the nutrient experiment (3 nutrient levels, 10 replicates per level) and another 30 plant cuttings were used in the dissolved oxygen experiment (3 dissolved oxygen levels, 10 replicates per level), respectively. Before flooding treatment, the stem height and total fresh weight of each selected plant cutting were measured. All plant cuttings (hereafter referred to as individual plants) did not have any roots before flooding treatment, i.e., all roots of the treated plants were aquatic roots formed on the stem nodes during the treatment. For brevity we will often refer to them as “roots” below.

In nutrient treatment experiment, three nutrient levels, 100, 50, and 5% strength standard Hoagland’s solution (The nutrient composition of the full strength solution in deionized (DI) water was (mM): 6.5 K^+^, 14.5 NO_3_^-^, 2 NH_4_^+^, 2 Mg^2+^, 4 Ca^2+^, 2 H_2_PO_4_^-^, 2 SO_4_^2-^, 0.0046 BO_3_^3-^, 0.0005 Mn^2+^, 0.045 Cl^-^, 0.0002 Zn^2+^, 0.0001 MoO_4_^2-^, 0.0002 Cu^2+^, 0.045 Fe^3+^. The solution was adjusted to pH 6.5 using NaOH.) were applied for root-flooded *A. philoxeroides* plants. Each plant (viz. plant cutting) was put in a plastic bottle (volume: 1000 ml) which had been fully filled with Hoagland’s solution of one of the three strengths, part of the plant stem including four stem nodes was submerged in the nutrient solution. The bottle was covered by a lid which had two holes, one hole was used to let the stem of the plant pass through the lid and hold the plant upright during the experiment, the other hole was for introducing a tube into the nutrient solution to bubble air. The bottle was wrapped with aluminum foil thereby keeping the submerged stem part and the nutrient solution in darkness, and then placed in a growth chamber under the condition of 12 h light (ca. 280 μmol m^-2^ s^-1^, 25.0°C) and 12 h dark (25.0°C). Ten replicate plants were randomly assigned to each nutrient level. For each plant, the nutrient solution was renewed every 2 days until the end of the experiment. During the experiment, all bottles were gently bubbled with air to keep the nutrient solution saturated with air. The partial pressures of oxygen in the nutrient solutions were 20.5 ± 0.053 kPa (measured by using an oxygen microelectrode, tip diameter = 50 μm, OX50, Unisense, Denmark), the pH of the solutions was 6.5 ± 0.11 during the experiment. All plants were randomly repositioned within the growth chamber once a day.

In dissolved oxygen treatment experiment, *A. philoxeroides* plants were randomly selected and put in 100% strength standard Hoagland’s solutions with different dissolved oxygen levels. Three dissolved oxygen levels (100, 50, and 5% air saturation in Hoagland’s solutions) were set, which were equivalent to oxygen partial pressure of 20.6 ± 0.102, 10.2 ± 0.038, and 1.04 ± 0.029 kPa in solutions during the experiment, respectively. The dissolved oxygen levels were obtained by aerating the solutions with air/N_2_ mixtures containing 100, 50 and 5% air, respectively. Ten replicate plants were randomly assigned to each dissolved oxygen level, each of the plants was placed in a plastic bottle fully filled with Hoagland’s solution, with part of its stem including four stem nodes submerged. The pH of the solutions was 6.4 ± 0.09 during the experiment. The growth conditions including illumination and temperature, the dissolved oxygen monitoring of solution, the solution renewal frequency, and the plant repositioning frequency were the same as those in nutrient treatment experiment.

### Plant Measurements

Each experiment lasted 23 days after which all plants were harvested. Stem height, total fresh weight of plant, and fresh weight of all roots (produced during treatment) were measured. In addition, the root number (the number of first order roots), total root length, root surface area and root volume of each harvested plant were measured by a Root Analysis System (WinRhizo Pro.2004c, Regent, Canada). 2–3 roots with approximately same size (diameter) were randomly chosen from each harvested plant for root porosity and aerenchyma investigation in nutrient treatment as well as dissolved oxygen treatment. For each root, a free hand transverse section of the root was made at the position approximately 0.5–1 mm away from its attachment point to the stem, the transverse section area of the root and the total area of aerenchyma in the root were measured by using a stereo-microscope (SMZ25, Nikon, Japan) and software (NIS-elements Imaging Software, version 4.30), and the proportion of total area of aerenchyma to the transverse section area of the root was calculated to indicate root porosity. Moreover, the transverse section of each root was divided into four quadrants, rows of aerenchyma channels aligning from the central vascular cylinder to the epidermis of the root in each quadrant were randomly selected and the transverse section area, the longest and shortest diameter of each aerenchyma channel were measured (NIS-elements Imaging Software, version 4.30).

### Data Analysis

The relative growth rates of fresh weight (RGR_FW_) and stem height (RGR_H_) were calculated for each plant by using the following formula:

$$\text{RGR}(\text{g}\;{\text{g}}^{\text{-}}{}^{\text{1}}\;{\text{day}}^{\text{-}}{}^{\text{1}}\text{or}\;\text{cm}\;{\text{cm}}^{\text{-}}{}^{\text{1}}\;{\text{day}}^{\text{-}}{}^{\text{1}})\;\text{=}\;({\text{lnA}}_{\text{2}}\;{\text{-lnA}}_{\text{1}})\text{/}({\text{t}}_{\text{2}}\;{\text{-t}}_{\text{1}})$$

Where A_1_ and A_2_ refer to the fresh weight or stem height of each plant at the start and end of the treatments, respectively, and t_1_ and t_2_ refer to starting and ending time of the treatments, respectively.

Plant biomass allocation to roots (g g^-1^) was calculated as: root fresh weight increment/fresh weight increment of the whole plant. Ratio of root surface area to root volume (cm^2^ cm^-3^) of each plant was calculated as: root surface area/root volume. Linear regression analyses were performed to investigate the relationship between the root surface area and plant fresh weight for plants under each treatment level.

One-way ANOVAs were conducted to investigate the effects of the nutrient treatment as well as the dissolved oxygen treatment on the RGR_FW_, RGR_H_, biomass allocation to roots, root number, total root length, ratio of root surface area to root volume, root porosity, and size of aerenchyma channels in roots of plants. Logarithm transformation of data was done to equalize variance if necessary. Differences between treatment levels were detected using Duncan’s multiple range test, and significant differences were reported at *P* < 0.05. Linear regression between plant fresh weight and total root surface area was conducted for plants subjected to each of three nutrient levels as well as each of three dissolved oxygen levels. The difference between slopes of regression lines was investigated by stepwise regression, with plant fresh weight as dependent variable and total root surface area, treatment level, and the interaction between total root surface area and treatment level as independent variables. The independent variable: interaction between total root surface area and treatment level, was obtained by multiplying total root surface area with treatment level. If the interaction between total root surface area and treatment level was significant and needed to be incorporated into regression, this means that the slopes of regression lines were different. All analyses were performed using SPSS 21.

## Results

### Plant Growth and Biomass Allocation to Roots

**FIGURE 1:**
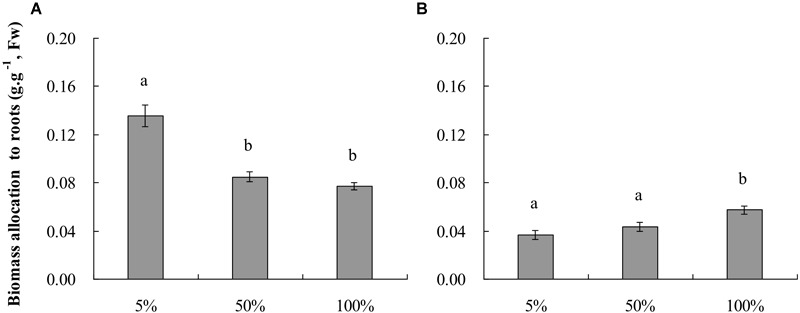
Plant biomass allocation to roots (means ± s.e., *n* = 10) of root-flooded *Alternanthera philoxeroides* subjected to nutrient **(A)** and dissolved oxygen **(B)** treatments. The set-up of nutrient and dissolved oxygen levels was the same as that in Table 1. Within each treatment, the significant difference between treatment levels is indicated by different letters (Duncan’s multiple range test, *P* < 0.05).

The growth of root-flooded *A. philoxeroides* plants was hampered by nutrient shortage in the water column (Table 1), the relative growth rates of plant fresh weight (RGR_FW_) and plant stem height (RGR_H_) of plants growing in 5% strength standard Hoagland’s solution were smaller than those of plants growing in 50 and 100% strength solutions. Plants in 50 and 100% strength solutions did not show a difference in RGR_FW_ or RGR_H_. With decreasing nutrient levels, *A. philoxeroides* tended to allocate proportionally more of its newly gained biomass to roots (Figure 1A); plants in 5% strength Hoagland’s solution had the largest biomass allocation to roots and plants in 100% strength solution the lowest.

**Table 1:** Relative growth rates of root-flooded *Alternanthera philoxeroides* in terms of plant fresh weight (RGR_FW_) and stem height (RGR_H_) under different nutrient and dissolved oxygen levels (means ± s.e., *n* = 10).

Treatment		RGR_FW_	RGR_H_
		**(mg g^-1^ day^-1^)**	**(cm cm^-1^ day^-1^)**
Nutrient level	5%	38.2 ± 1.48 ^a^	0.0161 ± 0.0008 ^a^
	50%	52.7 ± 2.24 ^b^	0.0211 ± 0.0013 ^b^
	100%	48.0 ± 2.26 ^b^	0.0202 ± 0.0010 ^b^
Dissolved oxygen level	5%	34.9 ± 3.79 ^a^	0.0190 ± 0.0011 ^a^
	50%	46.7 ± 1.51 ^b^	0.0229 ± 0.0013 ^b^
	100%	45.6 ± 2.85 ^b^	0.0235 ± 0.0010 ^b^

*Three nutrient levels (5, 50, and 100% strength of standard Hoagland’s solution saturated with air) and three dissolved oxygen levels (5, 50, and 100% air saturation in 100% strength of Hoagland’s solution) were applied. Values within each column with different superscripts are significantly different at *P* < 0.05.*

Dissolved oxygen concentration in water affected the growth of root-flooded *A. philoxeroides* even though the plants were growing in 100% strength Hoagland’s solutions and had good nutrient supply (Table 1). In this study, plants in solution with 5% air saturation (pO_2_ 1.04 ± 0.029 kPa) had smaller RGR_FW_ and RGR_H_ than plants in solutions with 50 and 100% air saturation (pO_2_ 10.2 ± 0.038 kPa and 20.6 ± 0.102 kPa, respectively); plants in solutions with 50 and 100% air saturation did not show significant differences in RGR_FW_ and RGR_H_ (Table 1). The biomass allocation of plants to roots increased significantly with increasing dissolved oxygen concentration in water; plants in solutions with 100% air saturation had the largest biomass allocation to roots (Figure 1B).

### Root Production, Root Morphology, and Root Efficiency

**FIGURE 2:**
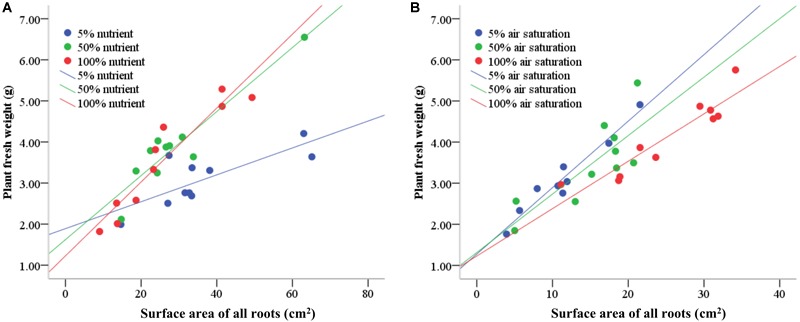
The relationship between plant fresh weight and total root surface area of root-flooded *A. philoxeroides* subjected to nutrient **(A)** and dissolved oxygen **(B)** treatments. The set-up of nutrient and dissolved oxygen levels was the same as that in Table 1.

Nutrient availability in water did not affect the number of roots produced on the submerged stem parts of *A. philoxeroides* plants, but had striking effects on the total root length and root surface area: volume ratio (Table 2). Plants in 5% strength Hoagland’s solution produced much longer roots than plants in 50 and 100% strength solutions. Furthermore, the roots produced were much more slender for plants in 5% than in 50 and 100% strength solutions, which was reflected by a larger ratio of root surface area to root volume (Table 2). The root efficiency of plants was influenced by nutrient supply, plants in 100% strength solution had the highest root efficiency (indicated by the steepest slope of the regression line of plant fresh weight plotted against total surface area of roots) while plants in 5% strength solution had the lowest root efficiency (Table 3 and Figure 2A). The slope of the regression line of plants in 5% strength Hoagland’s solution was significantly shallower than that of plants in 50 and 100% strength solutions (Table 3).

**Table 2:** Root number, total length of roots and ratio of root surface area to volume (A:V) of root-flooded *A. philoxeroides* under different nutrient and dissolved oxygen levels (mean ± s.e., *n* = 10).

Treatment		Number of first order roots (#)	Total length of roots (cm)	A:V (cm^2^ cm^-3^)
Nutrient level	5%	34.6 ± 4.1 ^a^	1183.2 ± 141.4 ^a^	301.7 ± 9.3 ^a^
	50%	31.4 ± 2.3 ^a^	722.3 ± 108.6 ^b^	206.2 ± 4.5 ^b^
	100%	32.4 ± 3.7 ^a^	646.1 ± 114.9 ^b^	207.0 ± 9.8 ^b^
Dissolved oxygen level	5%	31.9 ± 3.8 ^a^	269.0 ± 47.2 ^a^	235.6 ± 7.9 ^a^
	50%	31.4 ± 2.7 ^a^	499.2 ± 55.5 ^b^	263.1 ± 7.6 ^a^
	100%	44.8 ± 4.2 ^b^	628.6 ± 75.5 ^b^	201.1 ± 9.9 ^b^

*The set-up of nutrient and dissolved oxygen levels was the same as that in Table 1. Values within each column with different superscripts are significantly different at *P* < 0.05.*

**Table 3:** Equations and *R*^2^ of linear regression between plant fresh weight and total root surface area of root-flooded *A. philoxeroides* subjected to nutrient and dissolved oxygen treatments (*n* = 10).

Treatment		Regression equation	*R*^2^	Significance of difference between line slopes
Nutrient level	5%	Y = 0.033X + 1.89	0.61	a
	50%	Y = 0.078X + 1.62	0.87	b
	100%	Y = 0.090X + 1.23	0.89	b
Dissolved oxygen level	5%	Y = 0.162X + 1.28	0.94	a
	50%	Y = 0.142X + 1.32	0.65	a
	100%	Y = 0.115X + 1.23	0.87	b

*The set-up of nutrient and dissolved oxygen levels was the same as that in Table 1. The difference between slopes of regression lines was investigated by stepwise regression, with plant fresh weight as dependent variable and total root surface area, treatment level, and the interaction between total root surface area and treatment level as independent variables. The significance of difference between line slopes is indicated by different lowercase letters (*P* < 0.05).*

Dissolved oxygen concentration in water affected the root production as well as the root morphology of flooded *A. philoxeroides* plants (Table 2). Dissolved oxygen shortage in water reduced the number of roots produced on the plants (Table 2). Moreover, plants in solutions with 5% air saturation had the smallest total root length, while plants in solutions with 100% air saturation gained the largest total root length. Similar to how plants responded to nutrient shortage, roots produced on flooded plants became more slender upon dissolved oxygen shortage, as indicated by increased root surface area: volume ratio (Table 2). The root efficiency of plants increased with decreasing dissolved oxygen concentration in water. Plants in solutions with 5 and 100% air saturation presented the largest and the smallest root efficiency, respectively (Table 3 and Figure 2B). The slope of the regression line of plants in solutions with 100% air saturation was significantly smaller than that of plants in solutions with 5 and 50% air saturation (Table 3).

### Root Porosity and Root Aerenchyma Channel Size

**FIGURE 3:**
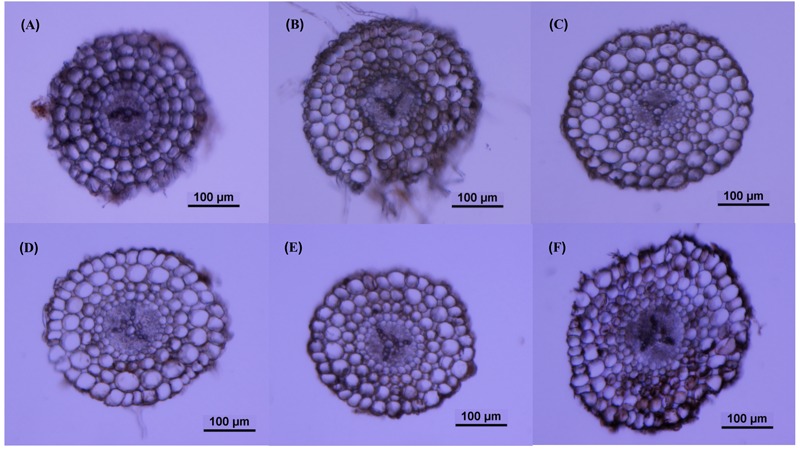
Typical root transverse sections of *A. philoxeroides* plants with roots flooded in 5% **(A)**, 50% **(B)** and 100% **(C)** strength Hoagland’s solutions saturated with air; and in 100% strength Hoagland’s solutions with 5% **(D)**, 50% **(E)** and 100% **(F)** air saturation.

**FIGURE 4:**
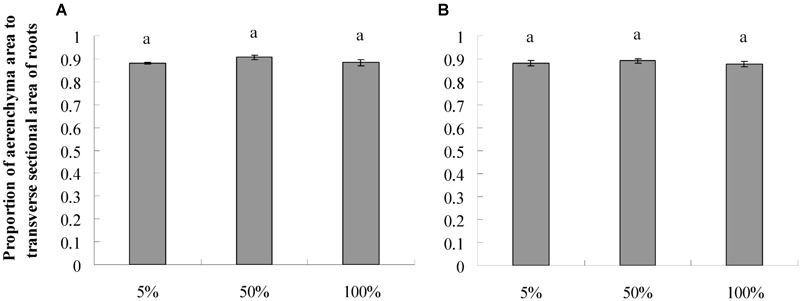
Root porosity (means ± s.e., *n* = 10) of root-flooded *A. philoxeroides* subjected to nutrient **(A)** and dissolved oxygen **(B)** treatments. Within each treatment, the significant difference between treatment levels is indicated by different letters (Duncan’s multiple range test, *P* < 0.05). The set-up of nutrient and dissolved oxygen levels was the same as that in Table 1.

**FIGURE 5:**
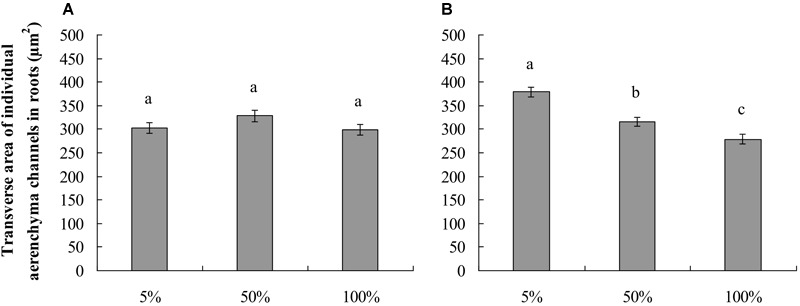
Lumen area of individual aerenchyma channels (means ± s.e., *n* = 10) in transverse section of roots produced by root-flooded *A. philoxeroides* subjected to nutrient **(A)** and dissolved oxygen **(B)** treatments. Within each treatment, the significant difference between treatment levels is indicated by different letters (Duncan’s multiple range test, *P* < 0.05). The set-up of nutrient and dissolved oxygen levels was the same as that in Table 1.

The roots produced on the submerged stem of *A. philoxeroides* had well-developed aerenchyma and exhibited high porosity in either nutrient or dissolved oxygen treatments. Honeycomb-shaped aerenchyma was formed in these roots everywhere except in the central vascular cylinder (Figure 3). Based on the analyses of transverse sections of roots, the nutrient level in water did not affect root porosity; the proportion of aerenchyma area to the whole area of the roots did not differ between plants flooded in 5, 50, and 100% strength Hoagland’s solutions (Figure 4A). Likewise, dissolved oxygen level in water did not affect the porosity of roots (Figure 4B). However, the effects of nutrient level and dissolved oxygen level on the size of aerenchyma channels in roots were different. The radius of aerenchyma channels in roots of plants flooded in 5, 50, and 100% strength Hoagland’s solutions were similar (Figure 5A), but the radius of aerenchyma channels in roots strongly decreased with an increase in dissolved oxygen concentration in water (Figure 5B). The roots of plants in solution with 5% air saturation had the biggest aerenchyma channels and the roots of plants in solution with 100% air saturation had the smallest aerenchyma channels (Figure 5B).

## Discussion

There have been previous notable examples of differential morphological or physiological responses of plants to deficiencies of different resources (ChapinIII, 1991). Among these responses, the important ones are those conducive to increasing resource uptake and efficiently utilizing limited resources (Hermans et al., 2006; Poorter et al., 2009). Our comparative study of responses of the terrestrial plant *A. philoxeroides* to nutrient and oxygen deficiency upon flooding adds a novel example to this literature.

### Plant Growth Impaired by Nutrient and Oxygen Shortage

We found that nutrient shortage impeded the growth of flooded *A. philoxeroides* (Table 1), as in previous studies (Hermans et al., 2006; Poorter et al., 2012). In this study, even at sufficient nutrient supply, we found that the growth of root-flooded *A. philoxeroides* was restricted by low dissolved oxygen level in water (Table 1). This result implies that, for root-flooded plants, even if oxygen could be transported from the aerial parts to the submerged parts of the plants, the transported amount of oxygen was insufficient to meet the demand, and the submerged parts still needed to absorb oxygen from the water column. Otherwise, the growth of root-flooded plants would not have been affected by the level of dissolved oxygen in water. Both nutrient and dissolved oxygen shortage clearly had an adverse effect on the growth of root-flooded *A. philoxeroides*. It is possible that nutrient shortage may have resulted in inadequate nutrient supply so that the plant growth was suppressed, and dissolved oxygen shortage could have weakened root nutrient absorption and impaired plant growth therefore, in spite of the sufficient nutrient supply in the water column.

### Responses of Plants to Nutrient Availability Were in Line With Expectations

Many studies have revealed that, when mineral nutrients are scarce in the environment, plants generally enhance root production and enlarge the root-environment interface to take up as many nutrients as possible for survival and growth (Hermans et al., 2006). In this study, with a decreasing nutrient availability in water, the plant biomass allocation to roots as well as total root length were increased, and the root diameter was reduced (as indicated by a larger ratio of root surface area to root volume), thereby providing plants with more roots and larger root-water contact to absorb nutrient ions (Figure 1 and Table 2). These results were similar to the responses of plants to low nutrient availability reported before (Marschner, 1998; López-Bucio et al., 2003; Hermans et al., 2006). Under low nutrient conditions, the quantity of nutrients absorbed per unit root surface area is generally decreased, and the contribution per unit root surface area to plant growth is lowered therefore. Our experimental results clearly supported the expectation that the root efficiency (viz. plant mass sustained based on per unit root surface area) of plants living in low nutrient environments should be smaller than that of plants having sufficient nutrient supply. This is illustrated by the significantly shallower slope of the linear relationship between plant fresh weight and total root surface area of *A. philoxeroides* in 5% strength Hoagland’s solution compared to that of plants in 50 and 100% strength solutions. Plants in 100% strength solution achieved the largest slope (Table 3 and Figure 2A).

### Responses of Plants to Dissolved Oxygen Availability Were Contrary to Expectations

In environments of low oxygen availability, aerobic respiration is restricted and an energy crisis usually occurs (Crawford and Braendle, 1996; Lambers et al., 2008), which then may impair the nutrient absorption capability of roots. It is reasonable to expect that plants in low oxygen environments would increase biomass allocation to roots, produce slenderer roots, gain larger root length, but exhibit lower root efficiency in terms of plant mass sustained per unit root surface area, like what plants respond to nutrient deficiency. In this study, similar to the plants’ response to low nutrient availability, *A. philoxeroides* plants subjected to dissolved oxygen deficiency produced slenderer roots (indicated by larger surface area: volume ratio) (Table 2). However, to our surprise, the responses of *A. philoxeroides* plants to dissolved oxygen deficiency in biomass allocation to roots, total root length, and root efficiency were completely contrary to the responses of the plants to nutrient deficiency. With decreasing dissolved oxygen levels, the biomass allocation to roots, root number, and total root length of *A. philoxeroides* plants were reduced, but the root efficiency was increased (Tables 2, 3 and Figure 1, 2). These results imply that, as far as the nutrient absorption capability per unit root surface area is concerned, the roots of plants experiencing low dissolved oxygen might have presented higher nutrient absorption capability than roots of plants in high dissolved oxygen environments. Consequently, the quantity of nutrients absorbed per unit root surface area of plants in low dissolved oxygen environments might have been increased and led to a larger root efficiency. The intriguing question is: what caused a larger root efficiency of *A. philoxeroides* plants in low dissolved oxygen environments?

In this study, we observed that neither nutrient level nor dissolved oxygen level affected the root porosity of *A. philoxeroides* (Figure 4). However, nutrient and dissolved oxygen treatments had different effects on the size of individual aerenchyma channels in roots. The diameter of aerenchyma channels was not affected by nutrient levels, but was enlarged significantly with decreasing dissolved oxygen levels (Figure 3, 5). According to Hagen–Poiseuille’s law, when gas moves through a conduit, the gas conductance is proportional to the fourth power of the conduit radius (Sutera, 1993; Lambers et al., 2008). For roots produced in low dissolved oxygen environments, it is very likely that they benefited from the wider root aerenchyma channels and get better internal oxygen supply. As a result, the nutrient absorption capabilities of roots on the basis of surface area could have been enhanced. This might be the reason why *A. philoxeroides* plants in low oxygen solutions gained higher root efficiency than they were supposed to behave. It should be pointed out based on our study that, although the growth of *A. philoxeroides* under low dissolved oxygen condition was hampered due to suppressed root production (in terms of both root number and root length) (Table 2), the plant still made the best of the bad situation, by forming big aerenchyma channels and producing roots of high surface area: volume ratio.

### Implications for Flooding Responses in the Field

Climate change will impact most ecosystems extensively (IPCC, 2012). Apart from other effects, climate change may cause the alteration of soil fertility and precipitation pattern, which are two important factors influencing the growth and behaviors of plants (especially terrestrial plants). It is predicted that the frequency and intensity of flooding events will increase due to mostly changed precipitation in near future (IPCC, 2012; Hirabayashi et al., 2013; Jongman et al., 2014), which implies that plants might have to confront flooding stress when they respond to nutrient environments. Previous studies revealed that aerenchyma played an important role in flooding tolerance of plants (Evans, 2003; Shimamura et al., 2003; Colmer and Voesenek, 2009; Yamauchi et al., 2013). However, these studies mainly focused on the porosity of aerenchymatous tissues and their overall function for flooding tolerance (Purnobasuki and Suzuki, 2004; Rich et al., 2012; Abiko and Obara, 2014). Based on our study, we found that only considering the porosity of plants’ tissues is not enough; the key role of the individual aerenchyma channel size, which we have preliminarily demonstrated in this study, should be taken into consideration in future studies on flooding tolerance of plants. Moreover, our findings demonstrate that, while the overall plant responses to comparable stress levels for nutrients and oxygen during flooding treatment were broadly comparable in terms of relative growth rate (Table 1), the mechanisms in terms of allocation and morphological response were completely different. To our knowledge this is the direct experimental demonstration how different agents of environmental stress related to flooding can induce fundamentally different but functionally beneficial plant responses.

We have realized through this study that plant biomass allocation to roots was not only affected by nutrient level in the environment, but also might be affected by nutrient absorption capability per unit root. The relation between biomass allocation to roots and nutrient level may be not as certain as it is usually supposed to be. In other words, higher nutrient availability in the environment does not necessarily lead to lower biomass allocation to roots, and conversely, lower nutrient availability in environments will not always result in higher biomass allocation to roots. When the nutrient absorption capability of roots is affected by factors such as aerenchyma morphology, oxygen availability, etc., the relation between plant biomass allocation to roots and nutrient level in environments may be changed. This alerts us that when we investigate the effects of nutrient supply on plant growth and biomass allocation, nutrient absorption capability of roots needs to be considered.

## Author Contributions

BZ conceived the original research plans and supervised the experiments. QA performed most of the experiments. KY, FL, and XZ provided assistance for some experiments. QA and BZ designed the experiments, analyzed the data, and wrote the article with contributions from PvB and JC.

## Conflict of Interest Statement

The authors declare that the research was conducted in the absence of any commercial or financial relationships that could be construed as a potential conflict of interest.
